# Independent predictors of survival in endometrium cancer: platelet-to-lymphocyte ratio and platelet/neutrophil/monocyte-to-lymphocyte ratio

**DOI:** 10.4274/jtgga.2017.0112

**Published:** 2018-06-04

**Authors:** Günsu Kimyon Cömert, Osman Türkmen, İrem Kar, Selcan Sınacı, Seval Yılmaz Ergani, Alper Karalök, Derman Başaran, Taner Turan

**Affiliations:** 1 Department of Gynecologic Oncology, University of Health Sciences, Etlik Zübeyde Hanım Women’s Diseases Training and Research Hospital, Ankara, Turkey; 2 Department of Biostatistics, Ankara University School of Medicine, Ankara, Turkey

**Keywords:** Endometrium cancer, platelet, monocyte, lymphocyte, neutrophil

## Abstract

**Objective:**

To evaluate the association between ratios of inflammatory markers and survival in endometrium cancer (EC).

**Material and Methods:**

Four hundred ninety-seven patients with epithelial EC were included. The evaluated ratios were neutrophil (N)/lymphocyte (L), neutrophil count divided by the lymphocyte count; platelet (P)/lymphocyte, platelets divided by the lymphocyte count; lymphocyte/monocyte (M), lymphocytes divided by the monocyte count; NM/L, neutrophil plus monocyte divided by the lymphocyte count; PNM/L, the sum total counts of platelets, neutrophils and monocytes divided by the lymphocyte count.

**Results:**

The median follow-up time was 24 months (1-129). Recurrence and exitus occurred in 34 (7%) and 18 (3.7%) patients, respectively. Metastasis in pelvic or para-aortic lymph nodes were significantly related only with low L/M. None of the inflammatory ratios were associated with disease-free survival. In multi-variant analysis, only high P/L (>168) and high PNM/L (>171) were related with a statistically significant hazard ratio for death of 2.91 (p=0.024) and 2.93 (p=0.023), respectively.

**Conclusion:**

The P/L and PNM/L were in relation with worse overall survival and also independent prognostic factors for OS.

## Introduction

The inflammatory response plays an important role in carcinogenesis and progression of cancer (1). A cancer- related inflammatory microenvironment can be reflected in the blood as measurable parameters. The basic changes are reported as a neutrophilia, thrombocytosis, and lymphocytopenia (1). Owing to challenges related with clinical adaptations of separate counts of lymphocytes, neutrophils, thrombocytes and monocytes, ratios of these inflammatory markers such as platelet-to-lymphocyte, neutrophil-to-lymphocyte and lymphocyte-to-monocyte are evaluated and have been used as prognostic factors in both infectious diseases and non-infectious diseases (2,3,4). In recent years, these rates have been clarified as having prognostic significance and survival prediction in a variety of solid cancers (5,6,7,8,9,10). Although the utility of these inflammatory parameters is easy and inexpensive, there is a paucity of data about the value of these ratios in gynecologic cancers, especially for endometrial cancer.

Endometrium cancer (EC) is the most common gynecologic cancer of the genital tract (11), but there is no distinct marker to predict pathologic findings and survival in EC. Therefore, the present study aimed to determine the association between ratios of complete blood counts and survival in EC.

## Material and Methods

Data of 497 patients with epithelial EC who underwent at least total abdominal hysterectomy and bilateral salpingo-oophorectomy between January 2005 and January 2016 in our clinic were reviewed whose results of complete blood counts were accessible. Data were obtained from the institution’s electronic database. The presence of secondary malignancy, having uterine sarcoma, and receiving neo-adjuvant chemotherapy were exclusion criteria of the study. Patients with any infectious disease or thromboembolism during the preoperative evaluation do not undergo elective surgery in our clinic. Accordingly, infectious and thromboembolism conditions were also excluded. Institutional review board approval was obtained from Etlik Zübeyde Hanım Women’s Diseases Training and Research Hospital before the study (2016; 206/16).

The surgical staging criteria of the International Federation of Gynecology and Obstetrics (2009) for EC (12) was used to determine the stage of disease. The largest tumor diameter in the uterus was accepted as the tumor size. Hematologic indices were calculated using an automated hematology analyzer system (ADVIA 2120, Siemens^®^ Healthcare, Germany). Preoperative complete blood counts including absolute count of leucocytes, neutrophils (N), lymphocytes (L), platelets (P) and monocytes (M) were collected. Parameters for ratios were constructed as follows: (i) N/L, neutrophil count divided by the lymphocyte count; (ii) P/L, platelet count divided by the lymphocyte count; (iii) L/M, lymphocyte count divided by the monocyte count; (iv) NM/L, neutrophil count plus monocyte count divided by the lymphocyte count and, (v) PNM/L, the sum total counts of platelets, neutrophils, and monocytes divided by the lymphocyte count.

Patients who had complete clinical response to their initial treatment were followed up with pelvic examinations and abdomen-pelvic ultrasonography quarterly in the first two years, semi-annually for up to five years, and annually thereafter. Annual chest X-rays and thoracic and/or abdominal computed tomography if needed were performed during the follow-up. Disease-free survival (DFS) was defined as the time interval from initial surgery to recurrence of disease. The period from surgery to death because of the disease (except in the first month after surgery) or last visit was defined as overall survival (OS).

Descriptive statistics are expressed as number/percentage for categorical variables and median (minimum-maximum) or mean ± standard deviation for continuous variables. The statistical significance of the demographic and clinic-pathologic parameters was evaluated using the chi-square test, Student’s t-test, and the Mann-Whitney U test. Survival on categorical variables was analyzed using the Kaplan–Meier method and the log-rank test was used to identify significant differences between groups. Multivariate analysis was performed using a Cox proportional hazards model that included variables (p-value <0.05) in the univariate analysis. The Statistical Package for the Social Sciences (SPSS version 11.5) was used in the analysis. P values less than 0.05 were considered to be statistically significant.

## Results

The median age of the entire cohort at diagnosis was 58 years (range, 29-92 years). Clinical and histopathologic findings, and values of complete blood counts of the entire cohort are shown in detail in Table 1. Adjuvant therapy was administered to 123 (25.7%) patients as a radiotherapy and/or chemotherapy. The mean time between analysis of the complete blood count and operation was 8±6 days. 

Non-endometrioid–type tumors and deep myometrial invasion were associated with significantly high P/L and high PNM/L. Advanced stage (≥ stage 2) and cervical stromal invasion were only related with low L/M. Although P/L, NM/L, N/L, and PNM/L were significantly high, L/M was significantly low in the presence of uterine serosal or ovarian involvement. P/L, NM/L, N/L and PNM/L were significantly high in the presence of lymphovascular space invasion (LVSI) and omental metastasis. The association between rates of complete blood counts and histopathologic findings are detailed in Table 2. 

Only L/M was associated with the presence of pelvic or para-aortic lymph node metastasis. There were statistically significant relations between low L/M and pelvic lymph node metastasis, and para-aortic lymph node metastasis. According to this finding, when the median value of L/M (5.46) was accepted as a cut-off value, low L/M (≤5.46) was significantly related with the presence of pelvic lymph node metastasis (p=0.031), but not related with para-aortic lymph node metastasis (p=0.087). 

The median follow-up time was 24 months (range, 1-129 months). Recurrence occurred in 34 (7%) patients during the follow-up period. The median recurrence time was 10 months (range, 1-56 months). Eighteen (3.7%) patients died of the disease. In all, 5-year DFS and 5-year OS were 86.5% and 94%, respectively. As shown in Table 3, non-endometrioid–type, advanced stage, high-grade, deep myometrial invasion, serosal involvement, cervical stromal invasion, LVSI, adnexal involvement, presence of lymph node metastasis, and omental metastasis were associated with worse DFS and OS. 

**Figure 1:**
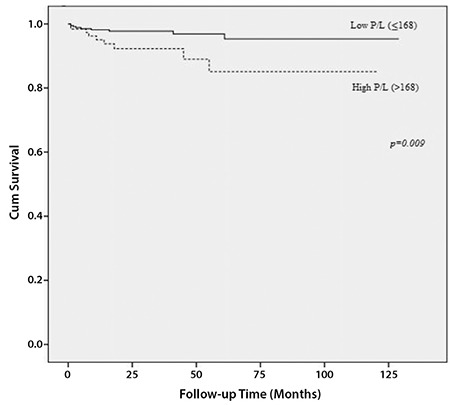
Association between overall survival and platelet-to-lymphocyte ratio *P/L: Platelet-to-lymphocyte ratio*

**Figure 2:**
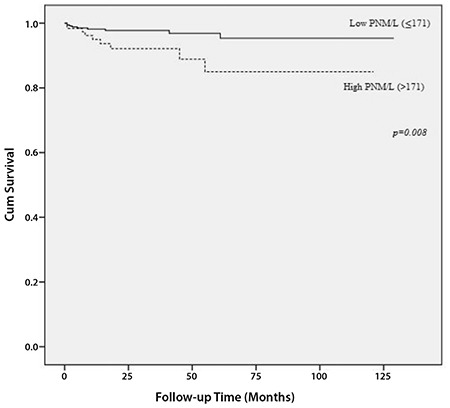
Association between overall survival and platelet/neutrophil/monocyte-to-lymphocyte ratio *PNM/L: Platelet/neutrophil/monocyte-to-lymphocyte ratio*

The cut-off value was determined as 168 for P/L, 171 for PNM/L, 2.23 for NM/L, 5.46 for L/M, and 2.06 for N/L as the best value to differentiate between patients’ survival in the entire cohort. Therefore, values were categorized as high and low levels according to their cut-off values. There were no statistically significant associations between preoperative ratios and DFS (Table 3). In the univariate analysis, both high preoperative P/L (>168) and PNM/L (>171) were significantly related with worse OS (Figure 1, 2). High P/L and PNM/L were related with a hazard ratio for death of 3.20 [95% CI: (1.27-8.07); p=0.014] and 3.25 [95% CI: (1.29-8.20); p=0.012], respectively (Table 3). According to these findings, because of the strong inter-relationship among the variables, two different models were created for multivariate analysis (Table 4). In the multivariate analysis, both high P/L (>168) and high PNM/L (>171) were related with a statistically significant hazard ratio for death of 2.91 [95% CI: (1.15-7.36); p=0.024] and 2.93 [95% CI: (1.16-7.40); p=0.023], respectively. 

## Discussion

The key findings of our study are that both high P/L and high PNM/L were significantly related with worse OS and independent prognostic factors for OS. However, none of the inflammatory ratios could predict DFS. 

Although EC is the most common gynecologic cancer, controversies continue with regard the extent of surgery, indications of lymphadenectomy, and criteria for the necessity of adjuvant therapy. Additionally, there are still no markers to give distinct prognostic information in EC. Intraoperative and postoperative pathology results are used to make decisions on those issues. Nevertheless, having a preoperative marker would provide more advantages such as increasing the accuracy of intraoperative decisions, avoiding overtreatment, preventing  unnecessary adjuvant therapy, and providing more accurate information to patients about the management of their disease and prognosis. 

Recent studies have focused on the prognostic role of the systemic manifestation of inflammatory cells in malignancies because one of the pathways of carcinogenesis is based on the inflammatory mechanism (1). The basic explanations for this argument are as follows; (1) neutrophilia and monocytosis are components of the proinflammatory process and are related with malignant cell proliferation, tumor-related angiogenesis and metastases, (2) thrombocytosis is explained by the paraneoplastic phenomenon that arises from tumor secretion of the proinflammatory cytokine interleukin-6, which increases thrombopoietin, but this mechanism is still not clear, (3) lymphocytes, which are an important component of host immunity, play a significant role in the anti-tumor immunologic reaction by inhibiting both proliferation and migration of tumor cells and inducing apoptosis (13,14). 

Lymphocytopenia, neutrophilia, thrombocytosis or monocytosis are associated with poor prognosis in endometrial cancer (15). However, the togetherness of these inflammatory parameters rather than a single effect of each of these is the important point in carcinogenesis. Therefore, recent studies have focused on the ratios of complete blood counts for prognostic information, prediction of pathologic features, and survival. Although Wang et al. (16) found that cervical stromal invasion in EC was significantly related with high values of both P/L and N/L, Haruma et al. (17) reported this association for only P/L. In addition, Haruma et al. (17) determined that deep myometrial invasion, advanced stage, ovarian metastasis, non-endometrial–type tumors were related with both high P/L and N/L. Only one study to date also evaluated L/M in EC; Cummings et al. (18) showed that although both high P/L and N/L were associated with advanced stage and LVSI, low L/M was only related with advanced stage. In spite of that, Kurtoglu et al. (19) reported that neither N/L nor P/L predicted stage or LVSI in EC. In our study, the significant associations between pathologic findings of EC and ratios were as follows; non-endometrioid–type tumor and deep myometrial invasion were associated with high P/L and PNM/L; advanced stage and cervical stromal invasion were only related with low L/M; presence of LVSI was associated with P/L, NM/L, N/L, and PNM/L; all ratios were related with uterine serosal and ovarian involvement.

Clinically, counts of these cells in the blood can have a role in the prediction of metastatic lymph nodes in EC. Matsuo et al. (20) found that elevated monocyte counts were significantly related with the presence of metastasis in pelvic lymph nodes in EC. Both P/L and N/L were found significantly high in the presence of lymph node metastasis in EC (17,18,21). Cummings et al. (18) determined that both P/L and N/L but not L/M were related with metastatic lymph nodes. In our study, only L/M was associated with metastatic pelvic or para-aortic lymph nodes. L/M was significantly low in patients with either pelvic or paraaortic lymph node metastasis. This finding can be accounted for by the important role of T-lymphocytes in inhibiting the migration of tumor cells and with the responsibility of neutrophils and especially monocytes in angiogenesis, but the role of thrombocytes is still not clear for tumor angiogenesis or migration (13,20).

The ratios, including complete blood counts, can also provide survival and prognostic information for solid tumors. High P/L, high N/L, and low L/M were found to be related with worse OS, cancer-specific survival (CSS) or DFS in solid tumors (7,22,23). A limited number of studies have discussed this issue in EC. Takahashi et al. (24) determined that elevated N/L was significantly associated with shorter OS in a univariate analysis, but no statistically significant relationship was found in multivariate analysis. Haruma et al. (17) evaluated the relation of P/L and N/L with survival and found that only P/L was an independent factor for OS and none of these was independently associated with DFS. Cummings et al. (18) reported that P/L and N/L but not L/M were independent prognostic factors for OS and CSS. Cut-off values that predicted survival varied between 150 and 300 for P/L and ranged from 2 to 5 for N/L (7,17,18,23). In our study, only high P/L and high PNM/L were associated with worse survival and both were independent prognostic factors for OS. None of the ratios was associated with DFS. The cut-off values of PNM/L and P/L for prediction of survival were 171 and 168, respectively.

The major limitation of our study is the retrospective design, which can cause difficulties in controlling for potential confounding factors. To the best our knowledge, the present study is the first to evaluate the relationship between PNM/L and survival for EC. Furthermore, a considerable number of patients with only epithelial endometrial cancer in a single center was evaluated in our study. 

In conclusion, the P/L and PNM/L ratios were associated with worse OS and also an independent prognostic factor for OS. However, there is a need for multi-center randomized controlled studies to make distinct conclusions. The togetherness of the inflammatory parameters has an important role in carcinogenesis. Therefore, future studies should focus on the role of combined ratios in EC and create a new risk model using ratios such as P/L and PNM/L.

## Figures and Tables

**Table 1:**
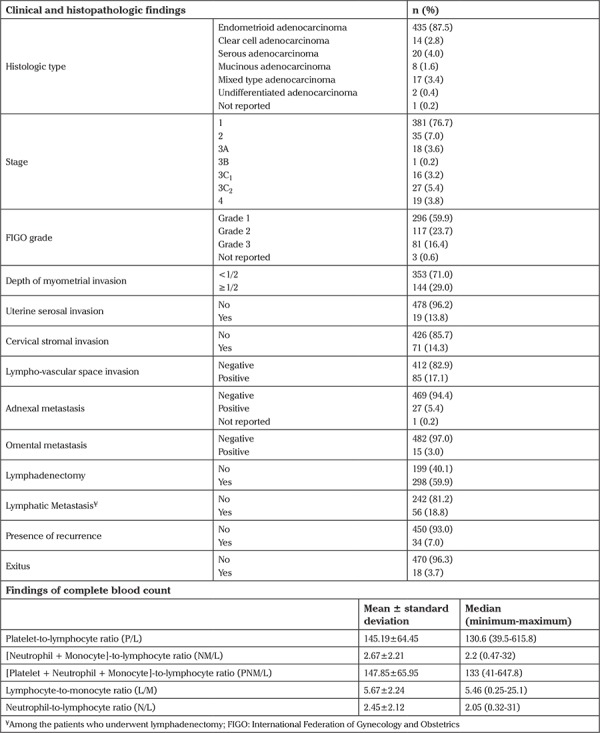
Findings of clinical, histopathologic, and complete blood count of the entire cohort

**Table 2:**
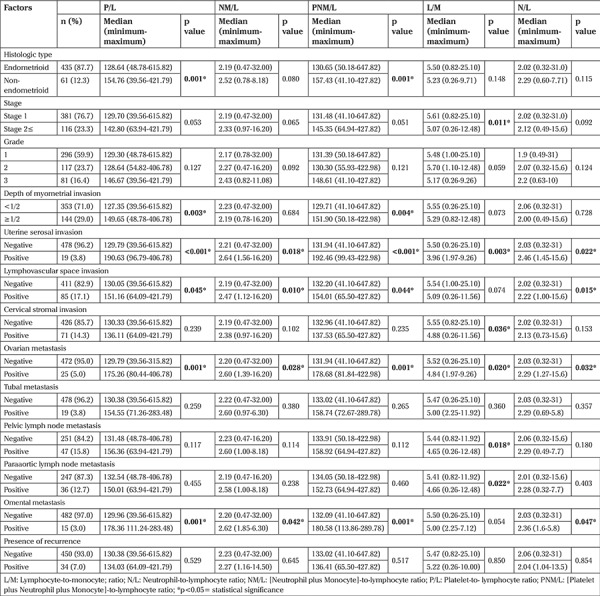
Associations between clinical-histopathologic features and ratios of complete blood counts

**Table 3:**
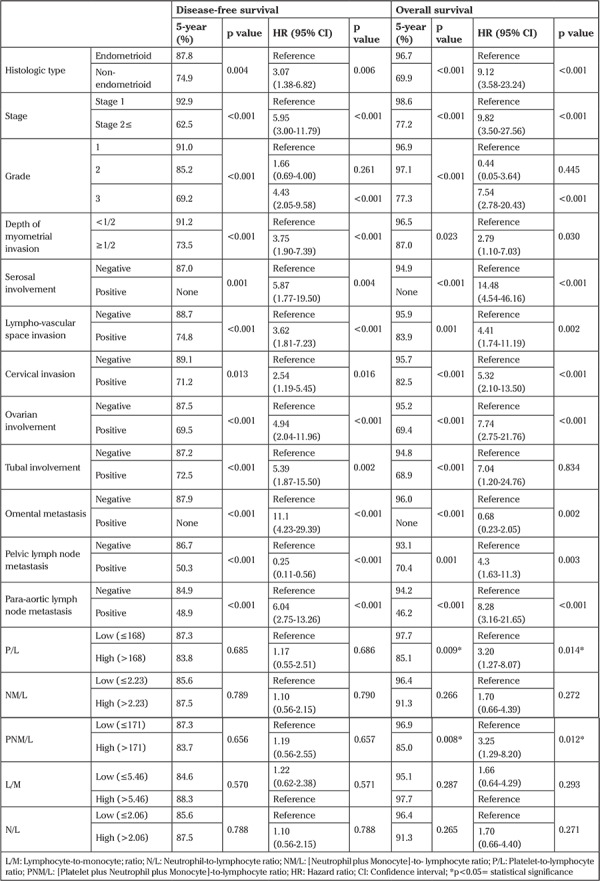
Univariate analysis of histopathologic features and ratios of complete blood counts for disease-free survival and overall survival

**Table 4:**
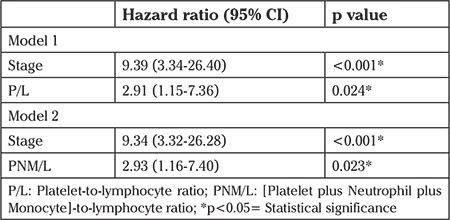
Multivariate analysis of P/L and PNM/L for overall survival

## References

[1] Elinav E, Nowarski R, Thaiss CA, Hu B, Jin C, Flavell RA (2013). Inflammation-induced cancer: crosstalk between tumours, immune cells and microorganisms. Nat Rev Cancer.

[2] Acet H, Ertas F, Akıl MA, Özyurtlu F, Polat N, Bilik MZ, et al (2016). Relationship Between Hematologic Indices and Global Registry of Acute Coronary Events Risk Score in Patients With ST-Segment Elevation Myocardial Infarction. Clin Appl Thromb Hemost.

[3] Kara A, Guven M, Yilmaz MS, Demir D, Elden H (2018). Are neutrophil, platelet and eosinophil-to-lymphocyte ratio and red blood cell distribution width can be used for nasal polyposis?. Eur Arch Otorhinolaryngol.

[4] Guclu M, Faruq Agan A (2017). Association of Severity of Helicobacter pylori Infection with Peripheral Blood Neutrophil to Lymphocyte Ratio and Mean Platelet Volume. Euroasian J Hepatogastroenterol.

[5] Njølstad TS, Engerud H, Werner HM, Salvesen HB, Trovik J (2013). Preoperative anemia, leukocytosis and thrombocytosis identify aggressive endometrial carcinomas. Gynecol Oncol.

[6] Li MX, Liu XM, Zhang XF, Zhang JF, Wang WL, Zhu Y, et al (2014). Prognostic role of neutrophil-to-lymphocyte ratio in colorectal cancer: a systematic review and meta-analysis. Int J Cancer.

[7] Templeton AJ, McNamara MG, Šeruga B, Vera-Badillo FE, Aneja P, Ocaña A, et al (2014). Prognostic Role of Neutrophil-to-Lymphocyte Ratio in Solid Tumors: A Systematic Review and Meta-Analysis. J Natl Cancer Inst.

[8] Kawata A, Une Y, Hosokawa M, Uchino J, Kobayashi H (1992). Tumor infiltrating lymphocytes and prognosis of hepatocellular carcinoma. Jpn J Clin Oncol.

[9] Cho H, Hur HW, Kim SW, Kim SH, Kim JH, Kim YT, et al (2009). Pre-treatment neutrophil to lymphocyte ratio is elevated in epithelial ovarian cancer and predicts survival after treatment. Cancer Immunol Immunother.

[10] Teng JJ, Zhang J, Zhang TY, Zhang S, Li BS (2016). Prognostic value of peripheral blood lymphocyte-to-monocyte ratio in patients with solid tumors: a meta-analysis. Onco Targets Ther.

[11] Ferlay J, Steliarova-Foucher E, Lortet-Tieulent J, Rosso S, Coebergh JW, Comber H, et al (2013). Cancer incidence and mortality patterns in Europe: estimates for 40 countries in 2012. Eur J Cancer.

[12] Pecorelli S (2009). Revised FIGO staging for carcinoma of the vulva, cervix, and endometrium. Int J Gynaecol Obstet.

[13] Mantovani A, Allavena P, Sica A, Balkwill F (2008). Cancer-related inflammation. Nature.

[14] Stone RL, Nick AM, McNeish IA, Balkwill F, Han HD, Bottsford-Miller J, et al (2012). Paraneoplastic thrombocytosis in ovarian cancer. N Engl J Med.

[15] Matsuo K, Hom MS, Moeini A, Machida H, Takeshima N, Roman LD, et al (2015). Significance of monocyte counts on tumor characteristics and survival outcome of women with endometrial cancer. Gynecol Oncol.

[16] Wang D, Yang JX, Cao DY, Wan XR, Feng FZ, Huang HF, et al (2013). Preoperative neutrophil-lymphocyte and platelet-lymphocyte ratios as independent predictors of cervical stromal involvement in surgically treated endometrioid adenocarcinoma. Onco Targets Ther.

[17] Haruma T, Nakamura K, Nishida T, Ogawa C, Kusumoto T, Seki N, et al (2015). Pre-treatment neutrophil to lymphocyte ratio is a predictor of prognosis in endometrial cancer. Anticancer Res.

[18] Cummings M, Merone L, Keeble C, Burland L, Grzelinski M, Sutton K, et al (2015). Preoperative neutrophil:lymphocyte and platelet: lymphocyte ratios predict endometrial cancer survival. Br J Cancer.

[19] Kurtoglu E, Kokcu A, Celik H, Sari S, Tosun M (2015). Platelet Indices May be Useful in Discrimination of Benign and Malign Endometrial Lesions, and Early and Advanced Stage Endometrial Cancer. Asian Pac J Cancer Prev.

[20] Matsuo K, Yessaian AA, Lin YG, Pham HQ, Muderspach LI, Liebman HA, et al (2013). Predictive model of venous thromboembolism in endometrial cancer. Gynecol Oncol.

[21] Suh DH, Kim HS, Chung HH, Kim JW, Park NH, Song Y, et al (2012). Pre-operative systemic inflammatory response markers in predicting lymph node metastasis in endometrioid endometrial adenocarcinoma. Eur J Obstet Gynecol Reprod Biol.

[22] Nishijima TF, Muss HB, Shachar SS, Tamura K, Takamatsu Y (2015). Prognostic value of lymphocyte-to-monocyte ratio in patients with solid tumors: A systematic review and meta-analysis. Cancer Treat Rev.

[23] Templeton AJ, Ace O, McNamara MG, Al-Mubarak M, Vera-Badillo FE, Hermanns T, et al (2014). Prognostic role of platelet to lymphocyte ratio in solid tumors: a systematic review and meta-analysis. Cancer Epidemiol Biomarkers Prev.

[24] Takahashi R, Mabuchi S, Kawano M, Sasano T, Matsumoto Y, Kuroda H, et al (2015). Prognostic significance of systemic neutrophil and leukocyte alterations in surgically treated endometrial cancer patients: a monoinstitutional study. Gynecol Oncol.

